# Growth performance, survivability and profitability of improved smallholder chicken genetics in Nigeria: A COVID-19 intervention study

**DOI:** 10.3389/fgene.2022.1033654

**Published:** 2023-01-04

**Authors:** Oladeji Bamidele, Oludayo Micheal Akinsola, Abdulmojeed Yakubu, Waheed Akinola Hassan, Uduak Emmanuel Ogundu, Tunde Adegoke Amole

**Affiliations:** ^1^ International Livestock Research Institute (ILRI), Ibadan, Nigeria; ^2^ Department of Biological Sciences, Kings University, Odeomu, Nigeria; ^3^ Department of Theriogenology and Production, University of Jos, Jos, Nigeria; ^4^ Department of Animal Science, Centre for Sustainable Agriculture and Rural Development, Faculty of Agriculture, Nasarawa State University, Keffi, Nigeria; ^5^ Department of Animal Science, Usmanu Danfodiyo University, Sokoto, Nigeria; ^6^ Department of Animal Science and Technology, Federal University of Technology, Owerri, Nigeria

**Keywords:** smallholder poultry, livelihoods, food security, COVID-19, Nigeria, improved chicken genetics

## Abstract

The impact of COVID-19 pandemic on smallholder farming households (SFH) includes increased poverty, and loss of livelihoods. Provision of livestock to SFH is a helpful intervention to mitigate this impact. This study provided a total of 150 smallholder poultry farmers, randomly selected from three states (Kebbi, Nasarawa, and Imo) in Nigeria, with ten 5-week-old chickens (mixed sexes) each, of either FUNAAB Alpha or Noiler chicken genetics. The improved, dual-purpose chickens were evaluated for growth performance (GP), survivability and profitability. The birds were managed under semi-scavenging production system. Body weight, mortality, and cost of production (COP) were recorded every 4 weeks until 21 weeks of age. Profitability was a function of the COP, and the selling price for live-birds (cocks). Body weight of Noiler (1,927 g) birds was not significantly (*p* > .05) higher than FUNAAB Alpha (1,792 g) at 21 weeks. Agroecology and genetics had significant (*p* < .05) effects on GP and survivability. Survivability of FUNAAB Alpha was higher (*p* < .05) than Noiler, with Nasarawa (81%–96%), having the highest (*p* < .0001) survival rate compared to Imo (62%–81%), and Kebbi (58%–75%). At 21 weeks, the number of cocks and hens differed significantly (*p* < .05) within the states (Imo: 2.4 ± .2 and 5.4 ± .3; Kebbi: 2.6 ± .2 and 5.5 ± .3; and Nasarawa: 2.9 ± .2 and 5.8 ± .3). Nasarawa (NGN 7,808; USD 19) ranked best for profitability, followed by Kebbi (NGN 6,545; USD 16) and Imo (NGN 5,875; USD 14). Overall, this study demonstrates that provision of improved chickens to vulnerable SFH in Nigeria holds great potential for economic growth, and resilience during emergencies, such as the COVID-19 pandemic.

## 1 Introduction

The COVID-19 pandemic has resulted in economic hardships to smallholder farming households in low-to-middle income countries (LMIC). It increased the risk of poverty among these farming populations who were already vulnerable to food insecurity and loss of livelihoods due to other environmental, and climate-related risks. In Nigeria, during the pandemic, there was a 31% decrease in average monthly income of smallholder poultry farmers (SPF) which resulted in a 28% increase in the number of SPF living in poverty (Bamidele and Amole, 2021). In a recent study, we showed that about half (49%) of the total number of SPF, living above the international poverty line prior to the pandemic, had been plunged into poverty within a 15-month period after the onset of COVID-19 (Bamidele and Amole, 2021). The changes to household income, food security, and poverty index occasioned by the pandemic highlight the significance of livestock, especially chickens to the socio-economic status of SPF.

Several measures have been proposed as interventions to support resource-poor and vulnerable smallholder livestock farmers in LMIC, some of which include: improved access to animal health services and markets, provision of feeds and water supplies, availability of livestock re-stocking options, and targeted cash transfers (Catley, 2020; FSC, 2020). These interventions were proposed to mitigate the impact of the pandemic on household livelihoods and food security as well as prevent the adoption of negative coping mechanisms by farmers through indiscriminate sale of livestock, use of inefficient restocking options, abuse of veterinary and human-labeled medicines (i.e., antibiotics), reduced consumption of animal-sourced foods, and depletion of emergency savings (Sitko et al., 2022; FSC, 2020). In LMIC, interventions involving the use of sustainable smallholder poultry are central to livelihoods’ sustenance, social and economic development (Attia et al., 2022). The introduction of improved dual-purpose chickens has been found to be suitable for backyard poultry production (Torres et al., 2019; Guni et al., 2021a). Birhanu et al. (2022) reported that the use of improved tropically adapted chicken breeds increased the production and productivity of birds in smallholder flocks in sub-Saharan Africa. This eventually paved way for the generation of more income, while contributing to food security, social and ecological resilience (Dumas et al., 2016; Kassa et al., 2021). These improved chicken breeds have also been reported to be more preferred than the indigenous (native, unimproved) chickens by smallholder farmers in terms of market-oriented performance indices (Yakubu et al., 2020; Birhanu et al., 2022).

In the current study, our intervention focused on the provision of two improved, dual-purpose (meat and eggs) chickens to SPF for re-stocking purposes, and as a source of food and income in Nigeria. The two chickens, FUNAAB Alpha and Noiler, before the advent of COVID-19 pandemic have been tested, both under on-station, and on-farm (scavenging and semi-scavenging) conditions, and identified as low-input-high-output, farmer-preferred genetics for dual-purpose functions (Ajayi et al., 2020; Bamidele et al., 2020; Yakubu et al., 2020). Also, the potential of these chickens for improving household livelihoods and food security have been reported (Alabi et al., 2020). Therefore, the objective of this study was to evaluate the growth performance, survivability, and profitability of the two chickens as intervention measures for SPF during the recovery phase of the COVID-19 pandemic in Nigeria.

## 2 Materials and methods

### 2.1 Description of study area

The study was conducted between June and November 2021 in three states of Nigeria: Kebbi (Sudan savanna/northern Guinea savanna), Nasarawa (southern Guinea savanna/derived savanna) and Imo (lowland rainforest/swamp). Each state represented a distinct agroecological zone with its features as described by Yakubu et al. (2020), and the locations of the three states within the respective agroecologies have been highlighted on the map of Nigeria by Bamidele and Amole (2021). The states were selected for the intervention study based on a previous impact assessment of COVID-19 on smallholder poultry households, in both the northern and southern regions of Nigeria (Bamidele and Amole, 2021).

### 2.2 Sampling procedure

A total of 150 farmers were selected for the intervention. In each of the three states, two local government areas (LGA) were purposively selected from the list of LGAs that participated in the COVID-19 impact assessment study (baseline) (Bamidele and Amole, 2021). The selection of farmers within the LGAs was conducted at the village level. One village per LGA was randomly selected from the villages previously sampled during the baseline. Geolocations of the study sites are available at https://www.mapcustomizer.com/map/Nigeria_COVID-19_Intervention_study. From each village, 25 farmers were then selected, randomly, among the farmers who had been recruited into the baseline study. In total, 50 farmers were selected per state. The selection of farmers within the households was based on the persons (adults) primarily responsible for keeping the chickens. Also, all the farmers had not received any form of COVID-19 palliative from the Federal Government of Nigeria (FGN).

### 2.3 Animal distribution and husbandry practice

Ten pre-vaccinated 5-week-old chickens, of either FUNAAB Alpha or Noiler, were given to each of the farmers. During brooding, the chicks were vaccinated against Marek’s, Newcastle and Fowl pox diseases. The ten chickens were equivalent to 50% of NGN 20,000 COVID-19 cash transfer payments to the poorest of the poor by FGN (Channels tv, 2020; Erezi, 2020). The two chickens were distributed to the farmers using a simple random sampling technique as described by Ajayi et al. (2020). In each village, 10 farmers received FUNAAB Alpha chickens while 15 farmers received Noiler chickens. Prior to bird distribution, the chicks were tagged at the wing. The genetic composition of the two chicken groups have been described by Adebambo et al. (2018), and Sanda et al. (2022). During the period of study, the farmers practiced semi-scavenging system of production with daily feed supplementation, and night-time shelter. The daily feed supplementation included household kitchen-waste (based on food patterns), agricultural by-products and plant parts that were locally available to the farmers. The husbandry practice included Newcastle disease vaccination (booster doses), and treatment of common poultry diseases by the farmer either through ethnoveterinary medicines or synthetic (pharmaceuticals) antibiotics. Consequently, based on the use or non-usage of synthetic antibiotics, the farmers were further classified into two groups.

### 2.4 Data collection

Data collection tool was designed using the web-based Google Forms App (docs.google.com/forms). All the field officers were trained on the use of the tool, and data was entered using smartphones. Each village had an assigned field officer who visited each of the households to provide technical support to the farmer on smallholder poultry husbandry, monitor the birds, and collect data. The household visits to the farmers were from the time the chicks were distributed, at 5 weeks up to 21 weeks of age. Data on growth performance, mortality, and cost of production (feed and drugs) were recorded every 4 weeks. The protocol for data collection was as described by Ajayi et al. (2020). Body weight (g) was taken using a digital weighing scale, and mortality was recorded by actual count of dead birds. At week 21, profitability was determined based on the total cost of production and the expected selling price for live-birds. Each farmer determined the appropriate selling price as guided by the prevailing market price. The decision to either sell the birds or slaughter for meat consumption was made by the farmers. During data collection, all COVID-19 safety protocols were adhered to by the field officers and farmers.

### 2.5 Statistical analysis

The collected data was assessed as spreadsheet (Microsoft Excel) through the Google Workspace and imported into R version 3.5.1 software (R Core Team, 2018) using the xlsx package (0.6.5 version). Imported data was wrangled by modifying the formats of some variables (e.g., Use of antibiotics, cost price, etc.) and removing errors such as “NaN,” and the letter ‘O’ in place of zero. Also, prior to statistical analyses, the data was visualized using boxplot, and all outliers were removed. Growth performance data were analyzed using unbalanced type-III three-way Analysis of variance (ANOVA) implemented in R car (version 3.0-2) package (Fox and Weisberg, 2011) to test the fixed effect of genetics, sex, antibiotics usage, agro-ecology as well as their interactions on production performance of birds. Significant differences were separated using Tukey test (α = 0.05) for multiple comparisons through R least square means (version 2.30-0) (Length, 2016), and R multcomp (version 1.4-10) (Hothorn et al., 2008) packages. The Cox proportional hazards regression analysis using R survival (version 2.42-3) (Therneau, 2015) and survminer (version 0.4.4) (Kassambara and Kosinski, 2016) packages were also used to investigate the effects of genetics, sex, antibiotics usage, and agro-ecology on the survival of birds. Significance of these factors was tested using Kaplan–Meier and log-rank tests. Hazard ratios were derived from Cox models. Proportional hazards assumed a non-significant relationship between scaled Schoenfeld residuals and time. All statistical analyses were performed in R version 3.5.1 (R Core Team, 2018). The dollar (USD) to naira (NGN) exchange rate used for the profitability analysis was USD 1 to NGN = 410.66 as listed by the Central Bank of Nigeria 2021 (https://www.cbn.gov.ng/rates/ExchRateByCurrency.asp).

### 2.6 Ethical standard

The animal study protocol was approved by the Institutional Review Board (or Ethics Committee) of International Livestock Research Institute (ILRI) (ILRI COVID-19 Project 03/2021). All the farmers provided informed consent prior to the start of the study.

## 3 Results

### 3.1 Sociodemographic of smallholder poultry farmers

**FIGURE 1:**
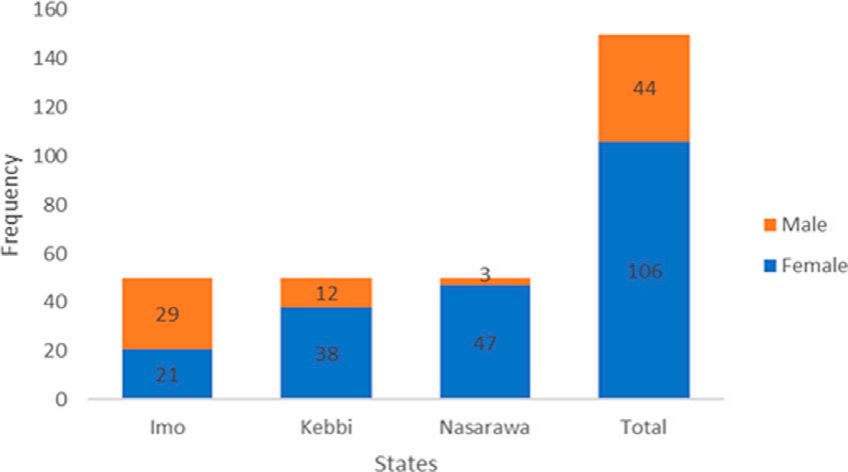
Gender distribution of smallholder poultry farmers in the study.

Figure 1 shows the gender distribution of farmers in this current study. Majority of the farmers were women (106, 70.7%), with Nasarawa (47, 94%) having the highest percentage of female farmers compared to Kebbi (76%), and Imo (42%) states. The average household size varied significantly (*p* < .05) between male and female smallholder poultry farmers in Imo (Male: 6.55 ± 1.73, female: 5.76 ± 1.66), Kebbi (Male: 10.80 ± 6.47, female: 6.47 ± 2.76) and Nasarawa (Male: 7.83 ± 1.69, female: 5.75 ± 1.95) states. The average household size was higher in Kebbi (8) than in Imo (6) and Nasarawa (6) states.

### 3.2 Growth performance and mortality of the improved chickens

Table 1 shows the effect of genetics on body weight, weight gain and mortality of the chickens. Genetic effect was similar (*p* > .05) for body weight, weight gain and mortality across the weeks. The results showed consistent increase in body weight from week 5–21. Noiler birds were heavier and gained more body weight than FUNAAB Alpha birds, at weeks 5, 13, 17, and 21, although these differences were not statistically significant (*p* > .05). At 9 weeks, bodyweight was lower (*p* > .05) in Noiler (626.16 g) compared to FUNAAB Alpha (629.39 g), and FUNAAB Alpha had a higher (*p* > .05) body weight gain (212.12 g) than Noiler (154.75 g). The mortality rate in FUNAAB Alpha ranged from 6%–18%, compared to 7%–25% in Noiler birds.

**TABLE 1:** Effect of genetics on body weight, weight gain, and mortality (LSM ± SE).

Week	Genetics	*N*	Body weight	CV (%)	Bodyweight gain	CV (%)	Mortality (%)
5	FUNAAB Alpha	600	414.17 ± 29.51	63.29	—	—	
	Noiler	900	471.72 ± 25.89	77.13	—	—	
9	FUNAAB Alpha	512	629.39 ± 16.54	32.08	215.12 ± 29.82	13.86	18.42 ± 5.23
	Noiler	744	626.16 ± 14.55	29.62	154.75 ± 26.23	16.95	25.19 ± 4.93
13	FUNAAB Alpha	477	960.82 ± 36.16	27.97	331.42 ± 32.31	70.94	8.14 ± 2.5
	Noiler	669	999.68 ± 31.81	46.04	373.52 ± 28.43	7.61	11.48 ± 2.5
17	FUNAAB Alpha	444	1,240.71 ± 48.71	32.78	279.93 ± 41.48	97.89	12.93 ± 4.2
	Noiler	629	1,322.11 ± 42.96	44.37	322.43 ± 36.58	11.35	6.99 ± 4.2
21	FUNAAB Alpha	418	1,792.38 ± 73.25	37.02	552.10 ± 49.98	90.32	5.76 ± 4.45
	Noiler	584	1,927.02 ± 65.48	54.72	604.98 ± 44.68	7.44	15.61 ± 4.2

*N*, number of birds; LSM ± SE, least-square means ± standard error; CV, coefficient of variation.

Body weights of the birds were significantly (*p* < .05) different across the agro-ecological zones, with Nasarawa State consistently having higher body weights compared to Kebbi and Imo states all through week 5–21 (Table 2). Agro-ecological zones had no significant (*p* > .05) effect on bird mortality, except at week 9 where mortality rate was significantly (*p* < .05) higher and similar in both Imo (26.7%) and Kebbi (35.4%), compared to Nasarawa (4.7%). At week 9 and 21, body weight gain was significantly different (*p* < .05) across the agro-ecological zones. At 9 weeks, birds in Imo (282.70 g) and Nasarawa (187.70 g) had 134.83 g and 39.83 g more body weight gain than Kebbi (147.87 g), respectively. On the other hand, at week 21, birds in Nasarawa significantly (*p* < .05) had the highest body weight (1,829.45 g) and body weight gain (447.36 g), compared to Imo (1,427.88 and 246.08 g) and Kebbi (1,588.28 and 206.75 g).

**TABLE 2:** Effect of agro-ecological zones on body weight, weight gain, and mortality (LSM ± SE).

Week	State	*N*	Body weight	CV (%)	Bodyweight gain	CV (%)	Mortality (%)
5	Imo	500	261.43 ± 38.45^b^	20.66			
	Kebbi	500	524.14 ± 38.45^a^	49.92			
	Nasarawa	500	569.06 ± 38.45^a^	78.85			
9	Imo	404	544.05 ± 38.84^c^	35.70	282.70 ± 22.34^a^	65.69	26.7 ± 5.21^b^
	Kebbi	373	672.01 ± 42.72^b^	29.43	147.87 ± 24.57^b^	28.06	35.43 ± 4.76^b^
	Nasarawa	479	756.74 ± 41.96^a^	18.32	187.70 ± 24.13^ab^	19.90	4.66 ± 4.76^a^
13	Imo	356	839.12 ± 39.87^c^	41.65	295.69 ± 22.94	11.47	15.2 ± 2.92
	Kebbi	349	960.39 ± 40.31^b^	28.55	288.83 ± 23.18	67.83	7.75 ± 2.92
	Nasarawa	441	1,083.93 ± 39.04^a^	41.04	327.26 ± 22.46	9.38	6.49 ± 2.92
17	Imo	335	1,181.80 ± 40.53^b^	49.41	342.961 ± 23.31	13.65	16.17 ± 5.11
	Kebbi	331	1,381.63 ± 39.25^ab^	39.97	421.84 ± 22.69	13.72	4.89 ± 5.11
	Nasarawa	407	1,382.32 ± 39.25^a^	31.57	298.48 ± 22.57	19.85	8.82 ± 5.11
21	Imo	310	1,427.88 ± 41.70^b^	48.83	246.08 ± 23.99^b^	21.03	16.39 ± 5.43
	Kebbi	288	1,588.28 ± 41.46^b^	40.26	206.75 ± 23.85^b^	16.75	16.37 ± 4.96
	Nasarawa	404	1829.45 ± 61.57^a^	7.10	447.36 ± 35.42^a^	66.42	1.07 ± 4.96

^abc^means within column sharing no common superscript were significantly different (*p* < .05).

*N*, number of birds; LSM ± SE, least-square means ± standard error; CV, coefficient of variation.

As shown in Supplementary Table S1, sexual dimorphism (*p* < .05) existed in both genetics for body weight as males were heavier than the female birds from week 9 through week 21. The coefficient of variations (CV) for bodyweight in female birds, ranged from 31% to 82% while CV for male birds ranged from 30% to 64%. Body weight gain were similar (*p* > .05) across the ages with the exception of week 17, where males (380.1 g) had over 70% increase (*p* < .05) in body weight gain than females (222.1 g). Across weeks 9–21, mortality was similar (*p* > .05) in both sexes, and ranged from 8.5% to 26.0% and 8.2%–18.0% for males and females, respectively.

Significant (*p* < .05) interaction effect of location, genetics and sex was evident on body weight at different ages (Table 3), although Noiler and FUNAAB Alpha birds in Nasarawa and Kebbi were superior (*p* < .05), compared to those in Imo. On the average, male birds of both genotypes were heavier (*p* < .05) than their female counterparts across the three different agro-ecological zones. With respect to body weight gain (Supplementary Table S2), FUNAAB Alpha and Noiler birds in Imo State had the highest (*p* < .05) body weight gain at 5–9 weeks of age and recorded the lowest gain in body weight at 17–21 weeks, compared to the other states. Male and female birds of both genotypes gained (*p* < .05) more body weight in Nasarawa than Imo and Kebbi at 17–21 weeks. Location, genetics and sex had no significant (*p* > .05) effect on body weight gain of TADP chickens at 9–13 weeks and 13–17 weeks of age, although male and female birds of both genotypes gained (*p* > .05) more body weight in Nasarawa than Imo and Kebbi.

**TABLE 3:** Effects of location, genetics and sex on body weight (g) of the chickens (LSM ± SE).

Location	Genetics	Sex	*N*	5 weeks	*N*	9 weeks	*N*	13 weeks	*N*	17 weeks	*N*	21 weeks
Imo	FUNAAB Alpha	F	103	206.1 ± 40.68^c^	80	501.93 ± 23.41^ij^	71	751.52 ± 49.99^e^	70	988.39 ± 67.36^i^	66	1,151.18 ± 101.64^j^
		M	97	247.11 ± 40.68^c^	73	578.93 ± 23.41^fgh^	69	849.13 ± 49.99^cde^	63	1,244.19 ± 67.7^bcdefgh^	60	1,525.77 ± 101.75^efgh^i
	Noiler	F	155	264.16 ± 37.47^c^	136	502.83 ± 21.56^hj^	117	789.08 ± 46.04^de^	113	1,068.91 ± 62.33^ghi^	103	1,281.5 ± 93.69^hij^
		M	145	305.17 ± 37.47^bc^	115	579.83 ± 21.72^egi^	99	886.7 ± 46.38^cde^	89	1,324.7 ± 63.47^abcdef^	81	1,656.09 ± 95.72^efg^
Kebbi	FUNAAB Alpha	F	91	468.8 ± 40.68^ab^	80	606.04 ± 23.39^fgh^	77	899.99 ± 49.94^cde^	73	1,122.65 ± 67.43^fhi^	69	1,354.49 ± 101.43^gij^
		M	109	509.81 ± 40.68^a^	86	683.04 ± 23.47^de^	82	997.6 ± 50.13^abcd^	78	1,378.44 ± 67.51^abcdeg^	65	1,729.08 ± 102.37^efh^
	Noiler	F	156	526.86 ± 37.47^a^	111	606.94 ± 21.62^egi^	100	937.56 ± 46.16^bcde^	93	1,203.16 ± 62.17^defghi^	84	1,484.81 ± 94.21^fghij^
		M	144	567.87 ± 37.47^a^	96	683.94 ± 21.87^df^	90	1,035.17 ± 46.7^abc^	87	1,458.95 ± 63.02^abc^	70	1859.4 ± 97.1^de^
Nasarawa	FUNAAB Alpha	F	104	513.73 ± 40.68^ab^	100	733.16 ± 23.43^cd^	92	1,063.04 ± 50.04^abc^	82	1,211.76 ± 67.56^cefghi^	81	2,243.66 ± 101.96^cd^
		M	96	554.74 ± 40.68^a^	93	810.16 ± 23.34^ab^	86	1,160.65 ± 49.85^ab^	78	1,467.55 ± 67.13^abd^	77	2,618.25 ± 100.89^ab^
	Noiler	F	157	571.78 ± 37.47^a^	150	734.06 ± 21.5^bd^	136	1,100.6 ± 45.93^abc^	128	1,292.28 ± 61.85^bcdefgh^	127	2,373.98 ± 92.97^bc^
		M	143	612.79 ± 37.47^a^	136	811.06 ± 21.57^ac^	127	1,198.22 ± 46.06^a^	119	1,548.07 ± 62.16^a^	119	2,748.57 ± 93.74^a^
Coefficient of variation				73.07		30.58		40.05		40.46		48.93
Source of variation (***p < .001,**p < .01, *p < .05)												
Location				***		***		***		***		***
Genetics				NS		NS		NS		NS		NS
Sex				NS		***		*		***		***
Interaction				***		***		***		***		***

^abcdefghij^means within column sharing no common superscript were significantly different (*p* < .05).

N, number of birds; LSM ± SE, least-square means ± standard error; NS, not significant.

At 9 weeks (Supplementary Table S3), higher mortality was evident for FUNAAB Alpha and Noiler birds across location, genetics and sex in Kebbi and Imo states compared to Nasarawa state. However, Mortality rate was similar (*p* > .05) at 13, 17, and 21 weeks, though Imo recorded the highest mortality rate, followed by Kebbi and Nasarawa.

As shown in Supplementary Table S4, antibiotics use was only significant (*p* < .05) on body weight and body weight gain at weeks 9 and 13, respectively. The mortality rate ranged from 7.6%–20.3%, and 4.5%–25.2%, for birds administered antibiotics and those not administered antibiotics, respectively. Birds with antibiotics usage had an incremental body weight gain from weeks 9–21 as against those not reared with antibiotics.

### 3.3 Survivability potential of the two chickens

**FIGURE 2:**
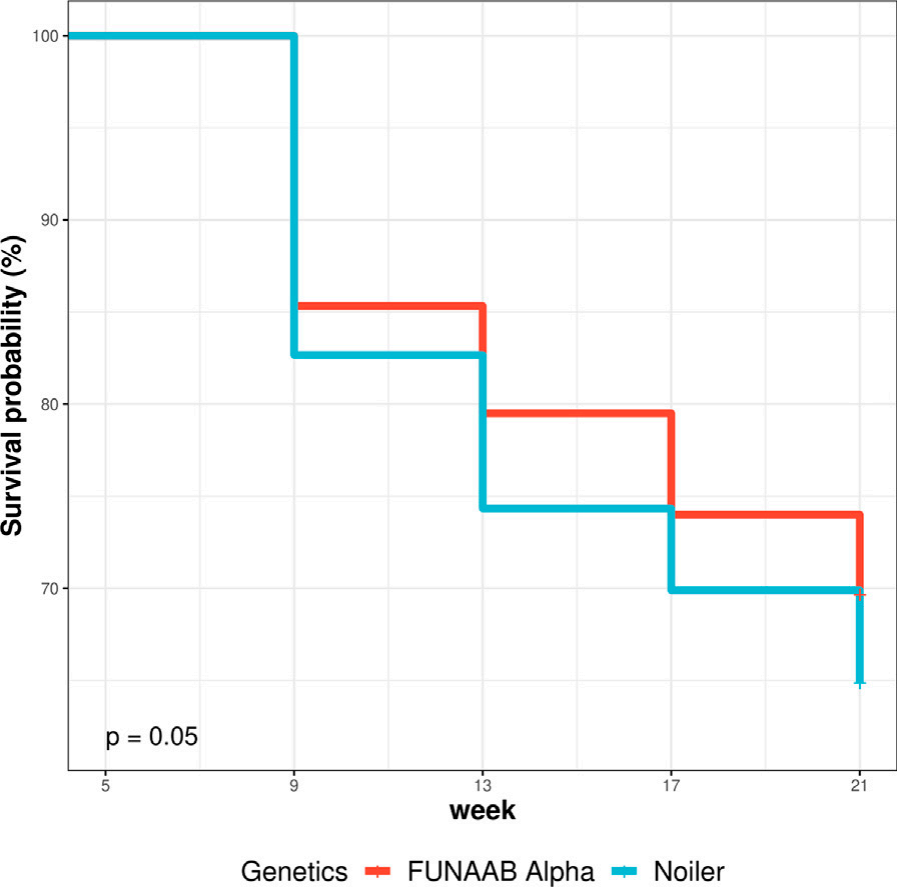
Effect of genetics on survival performance of birds (5–21 weeks).

**FIGURE 3:**
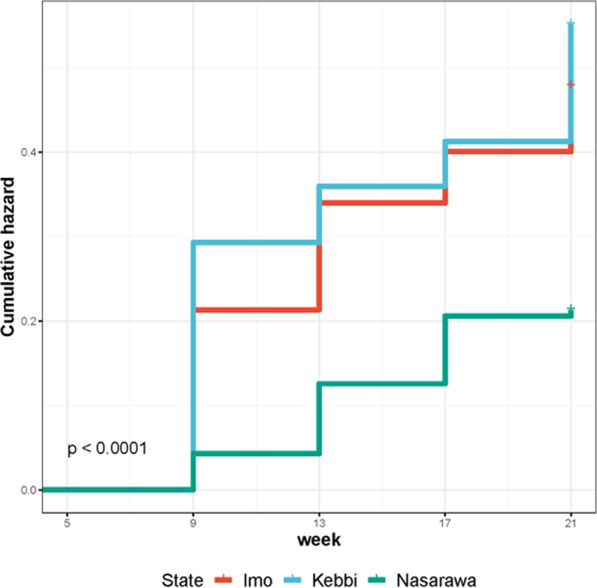
Effect of agro-ecological zone on the cumulative hazard of birds (5–21 weeks).

Genetics had a significant effect (*p* < .05) on the survival performance of birds and survivability decreases as the age of birds increases (Table 4). FUNAAB Alpha showed more survivability potential than Noiler birds by 3%, 7%, 6% and 7% at 9, 13, 17 and 21 weeks of age (Figure 2; Supplementary Figure S1), respectively. Noiler showed higher cumulative risk (.19–.43) of survival than FUNAAB Alpha (.16–.36) birds. The survival probability of the chickens was significantly (*p* < .001) influenced by agroecology (Table 5), with Nasarawa showing the lowest cumulative hazard (.04–.21) compared to Imo (.21–.48), and Kebbi (.29–.55). Birds reared in Kebbi showed the highest cumulative risk of survival (Figure 3; Supplementary Figure S2). The survival rate of the birds was highest in Nasarawa State (81%–96%), followed by Imo (62%–81%), and Kebbi (58%–75%) states.

**TABLE 4:** Effect of genetics on survival performance of birds (5–21 weeks).

Genetics	Week	IN	FN	NM	Surv. prob.± S.E	Cum. hazard ± S.E	Log-rank (*p*-value)
FUNAAB Alpha	9	600	512	88	.853 ± .017	.159 ± .017	.049
	13	512	477	35	.795 ± .021	.229 ± .021	
	17	477	444	33	.74 ± .024	.301 ± .024	
	21	444	418	26	.697 ± .027	.361 ± .027	
Noiler	9	900	744	156	.827 ± .015	.19 ± .015	
	13	744	669	75	.743 ± .02	.297 ± .02	
	17	669	629	40	.699 ± .022	.358 ± .022	
	21	629	584	45	.649 ± .025	.432 ± .025	

IN and FN, initial and final number of birds; NM, number of mortality; Surv. prob., survival probability; Cum. hazard, Cumulative hazard; S.E, standard error; Log-rank, Test of homogeneity for differences in survival.

**TABLE 5:** Effect of agro-ecological zone on survival performance of birds (5–21 weeks).

State	Week	IN	FN	NM	Surv. prob.± S.E	Cum. hazard ± S.E	Log-rank (*p*-value)
Imo	9	500	404	96	.808 ± .022	.213 ± .022	<.0001
	13	404	356	48	.712 ± .028	.34 ± .028	
	17	356	335	21	.67 ± .031	.4 ± .031	
	21	335	310	25	.62 ± .035	.478 ± .035	
Kebbi	9	500	373	127	.746 ± .026	.293 ± .026	
	13	373	349	24	.698 ± .029	.36 ± .029	
	17	349	331	18	.662 ± .032	.412 ± .032	
	21	331	288	43	.576 ± .038	.552 ± .038	
Nasarawa	9	500	479	21	.958 ± .009	.043 ± .009	
	13	479	441	38	.882 ± .016	.126 ± .016	
	17	441	407	34	.814 ± .021	.206 ± .021	
	21	407	404	3	.808 ± .022	.213 ± .022	

IN and FN, initial and final number of birds; NM, number of mortality; Surv. prob., survival probability; Cum. hazard, Cumulative hazard; S.E, standard error; Log-rank, Test of homogeneity for differences in survival.

**FIGURE 4:**
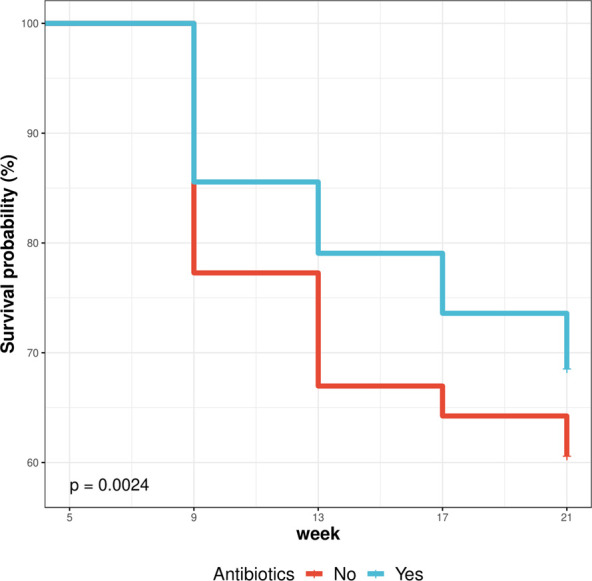
Effect of antibiotics usage on survival performance of birds (5–21 weeks).

Antibiotics usage had a significant (*p* < .01) effect on the survivability of the birds (Table 6). As shown in Figure 4, birds administered antibiotics showed higher propensity to survive (69%–86%) than those without antibiotics (61%–77%), consequently, a high cumulative hazard or risk of survival was observed in birds reared without antibiotics (Supplementary Figure S3).

**TABLE 6:** Effect of antibiotics usage on survival performance of birds (5–21 weeks).

Drug	Week	IN	FN	NM	Surv. prob.± S.E	Cum. hazard ± S.E	Log-rank (*p*-value)
No	9	330	255	75	.773 ± .03	.258 ± .03	.0024
Yes	9	1,170	1,001	169	.856 ± .012	.156 ± .012	
No	13	255	221	34	.67 ± .039	.401 ± .039	
Yes	13	1,001	925	76	.791 ± .015	.235 ± .015	
No	17	221	212	9	.642 ± .041	.443 ± .041	
Yes	17	925	861	64	.736 ± .018	.307 ± .018	
No	21	212	200	12	.606 ± .044	.501 ± .044	
Yes	21	861	802	59	.685 ± .02	.378 ± .02	

IN and FN, initial and final number of birds; NM, number of mortality; Surv. prob., survival probability; Cum. hazard, Cumulative hazard; S.E, standard error; Log-rank, Test of homogeneity for differences in survival.

### 3.4 Profitability of the smallholder chicken intervention

The effect of genetics on profitability of chickens is shown in Table 7. Profit per bird from the sales of FUNAAB Alpha (NGN 2,532; USD 6.2) and Noiler (NGN 2,388; USD 5.8) were not significantly different (*p* > .05). The same applies to cost of feed per household and cost of feed and drugs per bird. Also, the expected total profit was not significantly (*p* > 0.05) different between the two chickens.

**TABLE 7:** Effect of genetics on profitability (LSM ± SE) of the improved smallholder chicken intervention.

Trait	Genetics	Average no. of birds	CV (%)	LSM ± SE	CV (%)
Average cost of feed and drugs per HH	FUNAAB Alpha	10		7,091.77 ± 326.34	33.79
	Noiler	10		7,311.62 ± 287.66	31.64
Cost of feed and drugs per bird	FUNAAB Alpha	1		709.18 ± 32.63	33.79
	Noiler	1		731.16 ± 28.77	31.64
Expected sale per bird	FUNAAB Alpha	1		3,241.75 ± 125.62	28.92
	Noiler	1		3,119.54 ± 110.73	30.02
Expected profit per bird	FUNAAB Alpha	1		2,532.58 ± 110.43	32.58
	Noiler	1		2,388.37 ± 97.34	35.99
Expected total profit (males only at 21 weeks)	FUNAAB Alpha	2.84 ± .18	36.24	7,278.84 ± 618.37	50.69
	Noiler	2.43 ± .16	56.67	6,206.81 ± 545.07	76.36

LSM ± SE, least square means ± standard error; CV, coefficient of variation; HH, household.

Farmers in Imo and Nasarawa spent more (*p* < .001) on the total cost of feed and drugs per household, and cost of feed and drugs per bird. However, profit per bird was highest (*p* < .05) in Nasarawa and lowest in Imo and Kebbi (Table 8). Expected total profit from sale of male (cock) birds was highest in Nasarawa (NGN 780; USD 19.0), followed by Kebbi (NGN 6,544; USD 15.9) and Imo (NGN 5,875; USD 14.3), although these were not significantly (*p* > .05) different. At week 21, there were no significant differences (*p* > .05) in the average number of male (2.63 ± .15) and female (5.57 ± .26) birds between the agroecologies, however the number of cocks and hens differed significantly (*p* > .05) within the states (Imo: 2.41 ± .2 and 5.39 ± .29; Kebbi: 2.63 ± .19 and 5.53 ± .28; and Nasarawa: 2.86 ± .21 and 5.80 ± .31).

**TABLE 8:** Effect of agro-ecological zone on profitability (LSM ± SE) of the improved smallholder chicken intervention.

Trait	State	Average no. of birds	CV (%)	LSM ± SE	CV (%)
Average cost of feed and drugs per HH	Imo	10		7,860.74 ± 360.86^b^	34.86
	Kebbi	10		6,550.69 ± 345.38^a^	40.08
	Nasarawa	10		7,193.65 ± 384.57^ab^	17.41
Cost of feed and drugs per bird	Imo	1		786.07 ± 36.09^b^	34.86
	Kebbi	1		655.07 ± 34.54^a^	40.08
	Nasarawa	1		719.37 ± 38.46^ab^	17.41
Expected sale per bird	Imo	1		3,109.02 ± 138.9^ab^	33.39
	Kebbi	1		2,915.88 ± 132.94^a^	27.66
	Nasarawa	1		3,517.03 ± 148.03^b^	25.1
Expected profit per bird	Imo	1		2,322.95 ± 122.11^b^	39.32
	Kebbi	1		2,260.82 ± 116.87^b^	32.35
	Nasarawa	1		2,797.66 ± 130.13^a^	29.58
Expected total profit (males only at 21 weeks)	Imo	2.41 ± .2	56.69	5,875.89 ± 683.78	74.94
	Kebbi	2.63 ± .19	59.04	6,544.72 ± 654.45	86.28
	Nasarawa	2.86 ± .21	28.33	7,807.87 ± 728.71	34.25

^ab^means within column sharing no common superscript were significantly different (*p* < .05);

LSM ± SE, least square means ± standard error; CV, coefficient of variation; HH, household.

Farmers who reared their birds without antibiotics had the lowest (*p* < .05) cost of feed and drugs (Total and per bird) compared to those who administered antibiotics (Supplementary Table S5). The expected profit per bird, though higher in birds reared without antibiotics (NGN 2,577; USD 6.3) compared to those administered antibiotics (NGN 2,344; USD 5.7) was not significantly (*p* < .05) different in the two treatment groups. Supplementary Table S6 revealed that farmers in Imo, Kebbi and Nasarawa who reared FUNAAB Alpha and Noiler birds using antibiotics spent more (*p* < .05) on the cost of feed and drugs (Total and per bird). However, profit made from the sale of birds did not differ (*p* > .05) across genetics and agro-ecological zones in flocks with and without antibiotics usage.

Expected profit per bird, and sale per bird were the only variables significantly (*p* < .05) influenced by the interaction between gender and agro-ecological zone (Table 9). Although male and female farmers in Imo made more profit per bird (*p* < .05) than their Nasarawa and Kebbi counterparts, it did not reflect (*p* > .05) in the overall profit made. At 21 weeks, there were no significant differences (*p* > .05) in the average number of birds between the male and female farmers in Imo (5.54 ± .42 and 6.00 ± .22), Kebbi (6.40 ± .35 and 5.60 ± .17) and Nasarawa (5.67 ± .27 and 6.40 ± .23) states.

**TABLE 9:** Interaction effect of gender and location on profitability (LSM ± SE) of the improved smallholder chicken intervention.

Gender	Location	TCFD per HH	CFD per bird	EP per bird	ES per bird	ETP (males)
M	Imo	8,339.91 ± 417.17	833.99 ± 41.72	2,690.08 ± 113.97^a^	3,486.94 ± 216.22^a^	8,046.3 ± 1,071.09
F		8,132.38 ± 441.07	813.24 ± 44.11	2,687.35 ± 191.75^ab^	3,468.92 ± 128.52^ab^	8,020.64 ± 636.64
M	Nasarawa	7,995.88 ± 578.72	799.59 ± 57.87	2,254.09 ± 146.14^b^	3,085.34 ± 155.87^b^	6,601.7 ± 943
F		7,788.35 ± 343.98	778.83 ± 34.4	2,251.35 ± 138.22^b^	3,067.32 ± 164.8^b^	6,576.04 ± 671.44
M	Kebbi	6,993.72 ± 509.51	699.37 ± 50.95	2,205.06 ± 120.2^c^	2,901.69 ± 190.37^c^	5,999.26 ± 772.1
F		6,786.19 ± 362.78	678.62 ± 36.28	2,202.32 ± 168.81^c^	2,883.68 ± 135.55^c^	5,973.6 ± 816.33
Coefficient of variation		32.44	32.44	34.59	29.53	65.75
Source of variation (***p < .001,**p < .01, *p < .05)						
Gender		NS	NS	NS	NS	NS
Location		*	*	**	**	NS
Interaction		NS	NS	*	*	NS

^abc^means within column sharing no common superscript were significantly different (*p* < .05);

LSM±SE, least square means ± standard error; M, male; F, female; TCFD, total cost of feed and drugs; CFD, cost of feed and drugs; EP, expected profit; ES, expected sale; ETP, expected total profit; HH, household; NS, not significant.

## 4 Discussion

### 4.1 Sociodemographic characteristics of the farmers

The sociodemographic characteristics of farmers in this study are similar to that previously reported, before and during the COVID-19 pandemic, by Bamidele and Amole (2021). Majority of the households had women as the primary keepers of the flock. This is in consonance with several studies on the role of women in smallholder poultry production in sub-Saharan Africa, and in particular Nigeria (Birhanu et al., 2021; Alabi et al., 2020; Gueye, 2000; Wong et al., 2017; Alemayehu et al., 2018) and in sub-Saharan Africa.

### 4.2 Growth performance and mortality of birds

Studies on growth performance in indigenous breeds and varieties of chicken are increasingly receiving attention (González Ariza et al., 2021). Knowledge of the growth of animals is useful to improve management as well as feeding practices (Nguyen Hoang et al., 2021). Available reports in literature have shown that the genetic make-up of various animal breeds is an influential factor, which dominantly affect phenotypic characters (Buzala and Janicki, 2016; Nematbakhsh et al., 2021). In chickens, it has also been reported that genetic selection geared towards improvement in production traits could have an effect on the growth performance of the birds (Nematbakhsh et al., 2021). Both FUNAAB Alpha and Noiler have been genetically selected for improved dual-purpose (meat and eggs) performance in flocks owned by rural and peri-urban households. However, their body weight and weight gain performance appeared similar in the current study all through the growth phase (5–21 weeks). This is contrary to earlier findings (pre-COVID-19) on the same chicken genetics under similar environmental conditions, where the 18-week body weight (1,461.28 vs. 1,202.63 g) of Noiler birds was significantly higher than those of FUNAAB Alpha. The optimal performance of the birds could have been restricted as a result of certain nutritional limitations occasioned by the outbreak of COVID-19. Nutrition is a veritable component for the development of smallholder poultry, as it interacts with the genetics of the birds (Bamidele et al., 2020; Birhanu et al., 2022).

The feeds of birds under semi-intensive system of production are normally supplemented by the farmers (Tolasa, 2021). However, household food status and consumption patterns are some of the factors influencing the scavenging feed resource base available to chickens (Gondwe and Wollny, 2007). An earlier study revealed that COVID-19 had a negative effect on the average monthly income of farmers in the study area (Nigeria), where it was reduced from NGN 22,565 (USD 62.70) to NGN15,617 (USD 38.10) (Bamidele and Amole, 2021). This could have also reduced the ability of the farmers to supplement feeds quantitatively and qualitatively, with concomitant effect on the body size of the birds. Similar reports on the negative effects of the COVID-19 pandemic on avian species and the poultry industry generally have been documented (Esiegwu and Ejike, 2021; Seress et al., 2021). It is possible that Noiler birds will exhibit optimality under improved feeding conditions. However, the mature body weights (1,927.02 and 1,792.38 g) obtained in this study for Noiler and FUNAAB Alpha birds were higher than the values of 813.75 g, 1,400–1,660 g, 1,451–1716 g reported for mature indigenous chickens in Nigeria (Ajayi et al., 2020), Kenya (Mujyambere et al., 2022), and Algeria (Dahloum et al., 2016), respectively. Mortality rate did not differ between the two genotypes. This further confirms the potential of both tropically improved indigenous chicken genotypes to thrive in the smallholder production systems under the prevailing circumstances in Nigeria. It is congruous with the reports on the better performance of tropical breeds of chicken (Abegaz et al., 2019; Bamidele et al., 2020; Itafa et al., 2021; Kassa et al., 2021), which were primary developed for the improvement of genetics of growth among others (Zhang et al., 2020; Chomchuen et al., 2022).

The agro-ecology of the birds affected their body weight and weight gain performance. This could be attributed to varying environmental conditions, available scavenging resources, feed supplementation and managerial ability of the farmers in the different zones. Imo State is located in the wetter tropical rain forest zone of southern Nigeria while Nasarawa and Kebbi states are located in the hotter southern Guinea and Sahel savanna zones of northern Nigeria, respectively. Naturally, one would have expected birds in the rain forest zone to exhibit better performance. However, the reverse was the case in the current study, which was carried out from June–November 2021. This, probably, could be as a result of the fact that the stage of active growth of the birds coincided with the period of peak rainfall in the southern Guinea and Sahel savanna zones (between August and September) compared to the rain forest zone (July) (Ogungbenro and Morakinyo, 2014). A cool atmosphere could improve the comfort, feed efficiency, and overall productivity of birds. In a related study, Sztandarski et al. (2021) found associations between weather conditions and performance of birds. Alemu et al. (2021) also reported that climate may lead to body weight differences in different strains of improved tropically adapted chickens.

Male birds were heavier than their female counterparts, which could be attributed to sexual size dimorphism (SSD). According to Székely et al. (2007), SSD in most avian species favors male birds. Such dimorphism could have resulted from differential sexual- and natural-selection pressures experienced by both sexes (Yakubu et al., 2022), or from adaptive selection pressures which is a reflection of the evolution of males and females towards fitness optima divergence (Sztepanacz and Houle, 2021). At the level of smallholder poultry, it is possible that SSD may be influenced by differential ecological and socio-biological traits exhibited by male and female birds (Alarcón et al., 2017). This is more noticeable during the competition of male animals for mates in the context of polygyny (Cassini, 2022). Also, the sex-specific gene-regulation of body weight QTL (Johnsson et al., 2018) could be responsible for the differential expression of this trait in males and females. This study’s findings are similar to those previously reported in poultry (Dahloum et al., 2016; Toalombo Vargas et al., 2019; Yakubu et al., 2022).

The location, genetics and sex interaction effect on body weight where Noiler and FUNAAB Alpha birds seemed to have performed better in Nasarawa and Kebbi could be a reflection of the prevailing environmental conditions, available resources, management and socio-cultural practices of the smallholder farmers. We tried to compare our present results with earlier findings, and observed a similar trend as regards FUNAAB Alpha only. In an 18-week on-farm trial, the average body weights of FUNAAB Alpha in Imo, Nasarawa and Kebbi were reported as 1,072.33, 1,145.30, and 1,502.35 g (males) and 934.57, 1,001.91, and 1,294.52 g (females) (Ajayi et al., 2020). Thus, it appears that FUNAAB Alpha birds are more suitable to the environmental settings in Kebbi and Nasarawa compared to Imo. The present findings are consistent with the submission of Kassa et al. (2021) that breed-environment interaction could influence the phenotypic expression of traits. Similar genotype by location interaction effect on growth traits has been documented in improved indigenous and tropically adapted chickens (Ajayi et al., 2020; Guni et al., 2021b). The lower mortality of birds in Nasarawa State might be attributed to better management practices, which is consistent with the findings of Alemu et al. (2021).

Although antibiotics have been reported to act as growth promoters (Plata et al., 2022; a practice which is now banned in many countries due to antimicrobial resistance concerns), there was no clear-cut pattern of the effect of antibiotics usage on the growth traits of birds in the current study. While body weight was higher in flocks where antibiotics were used at 9 weeks of rearing, body weight gain of flocks without the use of antibiotics was higher. The same could be said of percentage mortality as there was no distinct difference between the two treatment groups. It is possible that other feeding and health management strategies including environmental conditions could have shaped the pattern of expression of both body weight and body weight gain as well as the mortality rate of the birds (Silveira et al., 2014; Nakkazi et al., 2015; El-Sabrout et al., 2022). Also, within the smallholder poultry production system in Nigeria, farmers’ use of antibiotics is primarily for therapeutic purposes, and not growth promotion (Bamidele et al., 2022a). This study did not assess if farmers administering antibiotics observed the required withdrawal period prior to sale (Turcotte et al., 2020).

### 4.3 Survivability of birds

The fitness of an animal in the environment where it is being kept is a measure of survival. The probability to survive appears higher in FUNAAB Alpha than in Noiler birds. This can be attributed primarily to genetics and management practices. It has been established that there is a relationship between the genetic constitution of birds, and their ability to cope in a particular environment (Cheng and Muir, 2005; Peeters et al., 2012). Also, better nutrition and health management practices can make birds to survive within their rearing environments (Kalia et al., 2017; Bughio et al., 2021; Chebo et al., 2022; Mujyambere et al., 2022). The observed genetic effect on survivability is consistent with the findings of Ajayi et al. (2020). Similar observations have also been made on chickens in Nigeria (Ademola et al., 2020), Ethiopia (Kassa et al., 2021), and Tanzania (Guni et al., 2021b).

The ability to adapt to varying environmental conditions is one of the attributes of improved indigenous and tropically adapted chickens. This ability includes a strong and efficient immune response to pathogenic infection, which assists in their survival (Wondmeneh et al., 2015), and adaptability (Sankhyan and Thakur, 2018) in the smallholder production systems. When other factors such as productive abilities, choice of breeds and traits of preference (Yakubu et al., 2020) are put into consideration, the present information may guide improved chicken breeds distribution to farmers in different agro-ecologies in Nigeria. Such breed by agro-ecology interaction effect on survivability has also been advocated by Guni et al. (2021b) as a means of distributing improved chicken breeds for optimal performance.

Health-related problems cause chicken losses on farm, hence the inclusion of antibiotics either in the feed or water of birds to ameliorate health-related conditions. The use of antibiotics could have conferred some health advantages on birds in the current study. Such birds, according to Berghof et al. (2019), are more resilient and less susceptible to environmental perturbations (diseases inclusive). Disease prevention in poultry is imperative for survival of the birds (Sargeant et al., 2019; Aboah and Enahoro, 2022), while intensifying the small-scale systems (Wilson et al., 2022). However, the abuse of antibiotics is of public health concerns, as it can lead to the multiplication of bacteria that are antibiotic resistant (Hafez, and Attia, 2020; Bamidele and Amole., 2021; 2022b; Zalewska et al., 2021). There was differential response of the sexes of the chickens in the different agro-ecologies to the use of antibiotics. However, as an alternative to antibiotics, probiotics and prebiotics are now being recommended as beneficial additives (Al-Khalaifah, 2018). It was difficult to compare our results with others due to dearth of information in literature. However, Nkansa et al. (2020) and Chah et al. (2022) reported the use of antibiotics for prophylaxis or treatment by backyard poultry farmers in Ghana and Nigeria, respectively.

### 4.4 Profitability of smallholder dual-purpose chicken enterprise

Considering the prevailing economic situation in Nigeria, post COVID-19, the profit level of both FUNAAB Alpha and Noiler birds, is an indication that investment in smallholder backyard poultry business is lucrative, provided all required inputs (housing, vaccination, supplementary feeding) are supplied. This, apart from contributing to household income, can improve food security and livelihoods of the farmers, as a way of attaining the Sustainable Development Goal of ending hunger and malnutrition by the year 2030 (Fang et al., 2021). Importantly, it will make available nutritious diets in form of quality meat and eggs ((Nuriliani et al., 2022). In this study, the average selling price (USD 7.7) for both chicken genetics was higher than the value (USD 1.6–2.4) reported for indigenous birds in Kenya (Otiang et al., 2020). The variation in price may however be attributed to the breed of chicken, size of bird, production cost, purchasing power, or the demand for local and improved chickens. On the other hand, the average selling price in Nigerian Naira (NGN 3,181) was similar to that previously reported (NGN 3,350) by Alabi et al. (2020) for both FUNAAB Alpha and Noiler birds.

The average cost of production, per bird per farmer from 5–21 weeks of production was NGN 720.2 (USD 1.8), at approximately NGN 45 (USD 0.1) per week. Transferring this cost to buyers at an average market price of NGN 3,181 (USD 7.8) per live-bird, yields an average profit of NGN 2,461 (USD 6.0) per cock sold. The average number of cocks and hens per farmer at the end of the study was three and six, respectively. When all the cocks are sold, this provides an average household income of NGN 7,383 (USD 18.0) to each farmer. In practice, cocks are sold more often than hens to meet urgent financial needs of smallholder poultry households while hens are kept for eggs (sale and consumption) as well as for breeding purposes (Alabi et al., 2020). Therefore, in additional to the sale of cocks, the hens (6 birds per household) are a potential source of extra household income (average of NGN 40–70/USD .10–.17 per egg) through sale of eggs (average of three eggs per week over a 6-month laying period) when properly kept under on-farm management conditions (Ajayi et al., 2020; Alabi et al., 2020). Also, chickens (cocks and hens) contribute to the nutritional and dietary requirements for animal-sourced proteins within the households. This is significant as it reduces household expenditure on eggs and meat, consequently increasing household savings, and improving the purchasing power for other basic necessities (Udo et al., 2011; Alabi et al., 2020). Our findings show that irrespective of gender, chickens can support household livelihoods as well as improve food security for resource-poor smallholder farmers. This is consistent with the findings of Agwu et al. (2020) where there was no gender differential in the profit made by male and female chicken producers.

The expected total profit realized from the sales of birds in Nasarawa, Kebbi, and Imo implies that smallholder poultry farming is a veritable venture in the three agro-ecological zones. This is in consonance with earlier submission (Alabi et al., 2020). It has also been reported in Vietnam that small-scale chicken production, which is dependent on the size of the flock is profitable (Truong et al., 2021). To boost production and increase profit under smallholder settings in Sub-Saharan Africa and Asia, some models such as microfranchising, microfinancing, cooperative farming, enterprise development, and out-grower model (Beesabathuni et al., 2018) have been proposed.

Use of ethnoveterinary medicines by farmers in the current study led to a reduction in cost of production, although it did not reflect in the overall profit. The continuous use of antibiotics on-farm by the smallholder farmers might have been encouraged by the patron-client relationship between farmers and dealers on pharmaceutical products (Masud et al., 2020). However, where resources are highly limited, the use of proven and effective ethnoveterinary medicines (Alders et al., 2018; Jambwa et al., 2022) is highly encouraged, as it has the potential to cut down production cost with possible increase in profit. The interaction between location, genetics and antibiotics did not significantly influence profitability. This is a further confirmation of the fact that both FUNAAB Alpha and Noiler chickens can be successfully reared in the three agro-ecologies of Nigeria with or without the use of antibiotics. The economic importance of these two improved indigenous chicken genetics, together with other tropically adapted breeds, has been highlighted in a scaling readiness study as part of a genetic solution strategy in Sub-Saharan Africa for smallholder poultry production systems (Sartas et al., 2021).

## 5 Conclusion and policy implications

Overall, this study demonstrates that provision of livestock, at 50% of the value of the cash transfer initiated by the Federal Government of Nigeria, to vulnerable smallholder farming households during the recovery phase of the COVID-19 pandemic holds great potential for economic growth and resilience of rural and peri-urban communities in Nigeria. In addition to cash-based interventions, provision of improved, locally-adapted chicken genetics to the poorest of the poor will also serve as a quick means of mitigating the impact of the pandemic, prevent negative coping strategies, and offer immediate as well as sustainable long-term nutritional relief and livelihoods support. Since smallholder poultry is practiced by most rural and peri-urban households in Nigeria, where it accounts for about 50% of total household income, rural economic recovery strategies in a post COVID-19 era, should include a value-chain approach that maximizes the economic potentials and improves the overall efficiency within the production system. The FGN’s poverty reduction policies aimed at lifting one hundred million Nigerians out of poverty within a decade should consider adopting women-friendly, agricultural technologies, such as the low-input, high-output, dual-purpose chicken genetics, for socioeconomic development of peri-urban and rural communities.

## Data Availability

The raw data supporting the conclusion of this article will be made available by the authors, without undue reservation.

## Supplementary material

SUPPLEMENTARY FIGURE S1 Effect of genetics on the cumulative hazard of birds (5–21 weeks).

SUPPLEMENTARY FIGURE S2 Effect of agro-ecological zone on survival performance of birds (5–21 weeks).

SUPPLEMENTARY FIGURE S3 Effect of antibiotics usage on the cumulative hazard of birds (5–21 weeks).

SUPPLEMENTARY TABLE S1 Effect of sex on body weight, weight gain, and mortality.

SUPPLEMENTARY TABLE S2 Effects of location, genetics and sex on body weight gain of the chickens.

SUPPLEMENTARY TABLE S3 Effects of location, genetics and sex on mortality of the chickens.

SUPPLEMENTARY TABLE S4 Effect of antibiotics use on body weight, weight gain, and mortality of the chickens.

SUPPLEMENTARY TABLE S5 Effect of antibiotics usage on profitability of the improved smallholder chicken intervention.

SUPPLEMENTARY TABLE S6 Interaction of location, genetics and antibiotics usage on profitability of the improved smallholder chicken intervention.
