# Supplementary material for: Growth performance, survivability and profitability of improved smallholder chicken genetics in Nigeria: A COVID-19 intervention study
Source: Front Genet. 2023 Jan 4;13:1033654. doi: 10.3389/fgene.2022.1033654 (PMC9846064; doi:10.3389/fgene.2022.1033654)
Supplement: Supplementary file 1 [file Table5.pdf]

Table S5. Effect of antibiotics usage on profitability (LSM±SE) of the improved smallholder chicken intervention

Trait	Antibiotics usage	Average no. of birds	CV %	LSM±SE	CV %
Average cost of feed and drugs per HH	No	10		6470.24±417.95[a]	47.89
	Yes	10		7933.15±224.94[b]	27.16
Cost of feed and drugs per bird	No	1		647.02±41.79[a]	47.89
	Yes	1		793.32±22.49[b]	27.16
Expected profit per bird	No	1		2577.13±141.43	36.45
	Yes	1		2343.82±76.12	34.06
Expected sale per bird	No	1		3224.15±160.88	35.1
	Yes	1		3137.14±86.58	27.94
Expected total profit (males only at 21 weeks)	No	2.34±0.23[b]	61.51	6340.93±791.96	84.26
	Yes	2.93±0.12[a]	44.68	7144.72±426.23	60.97

LSM±SE = least-square means ± standard error; CV = coefficient of variation; [ab]means within column sharing no common superscript were significantly different ($P<0.05$); HH=household
