# Supplementary material for: Growth performance, survivability and profitability of improved smallholder chicken genetics in Nigeria: A COVID-19 intervention study
Source: Front Genet. 2023 Jan 4;13:1033654. doi: 10.3389/fgene.2022.1033654 (PMC9846064; doi:10.3389/fgene.2022.1033654)
Supplement: Supplementary file 2 [file Table4.pdf]

Table S4. Effect of antibiotics use on body weight, weight gain, and mortality (LSM±SE) of the chickens

Period (weeks)	Antibiotics usage	N	Body weight	CV %	Bodyweight gain	CV %	Mortality %
5	No	330	437.86±37.8	79.18			
	Yes	1170	448.03±20.21	71.5			
9	No	255	582.14±21.18[b]	38.83	144.19±38.19	25.75	25.21±6.09
	Yes	1001	673.41±11.39[a]	27.43	225.69±20.53	35.41	20.25±4.49
13	No	221	994.57±46.31	36.78	412.42±41.39[a]	76.18	14.29±2.95
	Yes	925	965.93±24.89	40.97	292.52±22.25[b]	11.20	7.57±2.09
17	No	212	1291.83±62.39	43.91	297.29±53.13	54.50	4.79±5.11
	Yes	861	1270.99±33.65	39.52	304.94±28.65	47.46	12.55±3.61
21	No	200	1903.66±94.87	56.98	604.54±64.74	62.06	4.46±5.15
	Yes	802	1815.74±50.63	46.75	545.34±34.55	10.77	14.53±3.8

N = number of birds; LSM±SE = least-square means ± standard error; CV = coefficient of variation; [ab]means within column sharing no common superscript were significantly different ($P<0.05$)
