# Supplementary material for: Growth performance, survivability and profitability of improved smallholder chicken genetics in Nigeria: A COVID-19 intervention study
Source: Front Genet. 2023 Jan 4;13:1033654. doi: 10.3389/fgene.2022.1033654 (PMC9846064; doi:10.3389/fgene.2022.1033654)
Supplement: Supplementary file 3 [file Table1.pdf]

Table S1. Effect of sex on body weight, weight gain, and mortality (LSM±SE)

Week	Sex	N	Body weight	CV %	Bodyweight gain	CV %	Mortality %
5	F	766	422.44±27.51	82			
	M	734	463.45±27.51	64.42			
9	F	657	589.02±15.43^{b}	30.54	166.43±27.81	70.46	18.0±5.05
	M	599	666.53±15.47^{a}	29.55	203.45±27.89	14.1	26.01±5.05
13	F	593	931.52±33.72^{b}	48.13	342.5±30.14	28.65	11.11±2.51
	M	553	1028.97±33.83^{a}	31.07	362.44±30.23	72.39	8.52±2.51
17	F	559	1153.6±45.45^{b}	39.13	222.14±38.7^{b}	20.50	8.91±4.25
	M	514	1409.21±45.71^{a}	39.02	380.09±38.92^{a}	95.72	11.01±4.25
21	F	530	1672.45±68.35^{b}	51.05	516.92±46.64	72.90	8.19±4.44
	M	472	2046.95±69.61^{a}	45.06	632.96±47.5	10.95	13.77±4.44

N = number of birds; LSM±SE = least-square means ± standard error; CV = coefficient of variation; ^{ab}means within column sharing no common superscript were significantly different (*P<0.05*)
