# Supplementary material for: Growth performance, survivability and profitability of improved smallholder chicken genetics in Nigeria: A COVID-19 intervention study
Source: Front Genet. 2023 Jan 4;13:1033654. doi: 10.3389/fgene.2022.1033654 (PMC9846064; doi:10.3389/fgene.2022.1033654)
Supplement: Supplementary file 5 [file Table2.pdf]

Table S2. Effects of location, genetics and sex on body weight gain (g) of the chickens (LSM±SE)

Location	Genetics	Sex	N	5-9 weeks	N	9-13 weeks	N	13-17 weeks	N	17-21 weeks
Imo	FUNAAB Alpha	F	80	295.76±41.45^{abd}	71	249.59±45.15	70	236.73±57.35	66	165.4±69.36^{b}
		M	73	332.32±41.45^{a}	69	270.21±45.14	63	394.62±57.64	60	281.51±69.44^{b}
	Noiler	F	136	239.08±38.18^{abcde}	117	286.25±41.58	113	279.44±53.07	103	212.97±63.93^{b}
		M	115	275.64±38.46^{abc}	99	306.87±41.88	89	437.32±54.04	81	329.08±65.32^{b}
Kebbi	FUNAAB Alpha	F	80	137.03±41.41^{cef}	77	293.95±45.1	73	222.95±57.41	69	230.57±69.21^{b}
		M	86	173.59±41.57^{bcdef}	82	314.56±45.27	78	380.83±57.48	65	346.68±69.86^{b}
	Noiler	F	111	80.35±38.28^{f}	100	330.61±41.69	93	265.65±52.93	84	278.13±64.29^{b}
		M	96	116.91±38.72^{def}	90	351.23±42.17	87	423.53±53.66	70	394.24±66.26^{b}
Nasarawa	FUNAAB Alpha	F	100	218.86±41.49^{abcdef}	92	329.87±45.19	82	149.02±57.52	81	1034.58±69.57^{a}
		M	93	255.42±41.34^{abcdef}	86	350.49±45.02	78	306.9±57.16	77	1150.69±68.85^{a}
	Noiler	F	150	162.18±38.08^{abcdef}	136	366.54±41.47	128	191.72±52.66	127	1082.15±63.44^{a}
		M	136	198.74±38.19^{abcdef}	127	387.16±41.6	119	349.6±52.92	119	1198.26±63.97^{a}
Coefficient of variation				54.53		12.85		40.41		13.81
Source of variation (*** P<0.001,** P<0.01, * P<0.05)										
Location				**		NS		NS		***
Genetics				NS		NS		NS		NS
Sex				NS		NS		**		NS
Interaction				***		NS		NS		***

N = number of birds; LSM±SE = least-square means ± standard error; ^{abcdef}means within column sharing no common superscript were significantly different (*P<0.05*), NS = Not significant.
