# Supplementary figures and images for: Growth performance, survivability and profitability of improved smallholder chicken genetics in Nigeria: A COVID-19 intervention study
Source: Front Genet. 2023 Jan 4;13:1033654. doi: 10.3389/fgene.2022.1033654 (PMC9846064; doi:10.3389/fgene.2022.1033654)

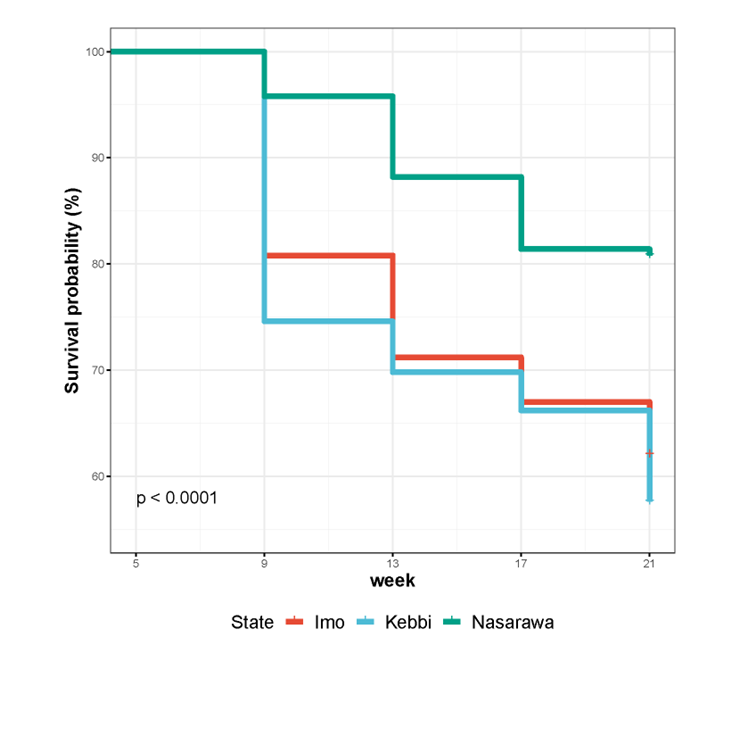

Supplement: Supplementary file 6 [file Image2.tif]
