# Supplementary material for: Growth performance, survivability and profitability of improved smallholder chicken genetics in Nigeria: A COVID-19 intervention study
Source: Front Genet. 2023 Jan 4;13:1033654. doi: 10.3389/fgene.2022.1033654 (PMC9846064; doi:10.3389/fgene.2022.1033654)
Supplement: Supplementary file 7 [file Table6.pdf]

Table S6. Interaction of location, genetics and antibiotics usage on profitability (LSM±SE) of the improved smallholder chicken intervention

Location	Genetics	Antibiotics usage	TCFD per HH	CFD per bird	EP per bird	ES per bird	ETP (males)
Imo	FUNAAB	No	7019.37±536.16[abcd]	701.94±53.62[abcd]	2511.7±181.43	3213.64±206.38	6010.01±1015.95
	Alpha	Yes	8482.28±430.71[ab]	848.23±43.07[ab]	2278.4±145.75	3126.62±165.79	6813.79±816.15
	Noiler	No	7239.21±526.16[abcd]	723.92±52.62[abcd]	2367.5±178.04	3091.42±202.53	4937.98±997.01
		Yes	8702.12±393.02[a]	870.21±39.3[a]	2134.19±132.99	3004.41±151.28	5741.77±744.72
Kebbi	FUNAAB	No	5709.31±513.54[cd]	570.93±51.35[cd]	2449.57±173.78	3020.5±197.67	6678.84±973.1
	Alpha	Yes	7172.22±440.46[abcd]	717.22±44.05[abcd]	2216.26±149.04	2933.49±169.54	7482.63±834.61
	Noiler	No	5929.16±496.48[bd]	592.92±49.65[bd]	2305.37±168	2898.28±191.1	5606.82±940.76
		Yes	7392.07±395.39[abcd]	739.21±39.54[abcd]	2072.06±133.79	2811.27±152.19	6410.6±749.21
Nasarawa	FUNAAB	No	6352.27±583.26[abcd]	635.23±58.33[abcd]	2986.42±197.37	3621.65±224.51	7941.98±1105.2
	Alpha	Yes	7815.18±412.63[ab]	781.52±41.26[ab]	2753.11±139.63	3534.63±158.83	8745.77±781.88
	Noiler	No	6572.12±572.23[abcd]	657.21±57.22[abcd]	2842.22±193.63	3499.43±220.26	6869.96±1084.3
		Yes	8035.03±370.25[ac]	803.5±37.02[ac]	2608.91±125.29	3412.41±142.52	7673.75±701.58
Coefficient of variation			32.44	32.44	34.59	29.53	65.75
Source of variation (*** P<0.001,** P<0.01, * P<0.05)							
Location			*	*	**	**	NS
Genetics			NS	NS	NS	NS	NS
Antibiotics			**	**	NS	NS	NS
Interaction			***	***	NS	NS	NS

LSM±SE = least square means ± standard error; [abcd]means within column sharing no common superscript were significantly different (*P<0.05*); TCFD =Total cost of feed and drugs; CFD = Cost of feed and drugs; EP =Expected profit; ES = Expected sale; ETP =Expected total profit; HH=household, NS = Not significant.
