# Supplementary material for: Growth performance, survivability and profitability of improved smallholder chicken genetics in Nigeria: A COVID-19 intervention study
Source: Front Genet. 2023 Jan 4;13:1033654. doi: 10.3389/fgene.2022.1033654 (PMC9846064; doi:10.3389/fgene.2022.1033654)
Supplement: Supplementary file 9 [file Table3.pdf]

Table S3. Effects of location, genetics and sex on mortality of the chickens (LSM±SE

Location	Genetics	Sex	N	9 weeks	N	13 weeks	N	17 weeks	N	21 weeks
Imo	FUNAAB Alpha	F	80	18.93±6.69^{b}	71	14.82±3.8	70	18.09±6.68	66	8.02±6.96
		M	73	26.94±6.69^{bc}	69	12.23±3.8	63	20.2±6.68	60	13.6±6.96
	Noiler	F	136	25.21±6.21^{bc}	117	18.16±3.8	113	12.15±6.68	103	17.32±6.46
		M	115	33.21±6.21^{bc}	99	15.57±3.8	89	14.25±6.68	81	22.9±6.46
Kebbi	FUNAAB Alpha	F	80	28.29±6.09^{bc}	77	7.37±3.8	73	6.81±6.68	69	8.94±6.33
		M	86	36.3±6.09^{b}	82	4.78±3.8	78	8.91±6.68	65	14.52±6.33
	Noiler	F	111	34.56±6.09^{bc}	100	10.71±3.8	93	0.86±6.68	84	18.23±6.33
		M	96	42.57±6.09c	90	8.12±3.8	87	2.97±6.68	70	23.81±6.33
Nasarawa	FUNAAB Alpha	F	100	2.48±6.09^{a}	92	6.12±3.8	82	10.74±6.68	81	6.37±6.33
		M	93	5.53±6.09^{ab}	86	3.53±3.8	78	12.84±6.68	77	0.79±6.33
	Noiler	F	150	3.79±6.09^{a}	136	9.46±3.8	128	4.79±6.68	127	2.93±6.33
		M	136	11.8±6.09^{ab}	127	6.87±3.8	119	6.9±6.68	119	8.51±6.33
Coefficient of variation				97.06		18.37		14.59		27.17
Source of variation (*** P<0.001,** P<0.01, * P<0.05)										
Location				***		NS		NS		NS
Genetics				NS		NS		NS		NS
Sex				NS		NS		NS		NS
Interaction				***		NS		NS		NS

N = number of birds; LSM±SE = least-square means ± standard error; ^{abc}means within column sharing no common superscript were significantly different (*P<0.05*), NS = Not significant.
